# Migration Mechanism of Chlorine during Hydrothermal Treatment of Rigid PVC Plastics

**DOI:** 10.3390/ma16175840

**Published:** 2023-08-25

**Authors:** Ling Zhang, Qing Wang, Faxing Xu, Zhenye Wang

**Affiliations:** 1 Engineering Research Centre of Oil Shale Comprehensive Utilization, Ministry of Education, Northeast Electric Power University, Jilin City 132012, China; 2 Jilin Institute of Chemical Technology, Jilin City 132022, China; 3 Jilin Feite Environmental Protection Co., Ltd., Jilin City 132200, China

**Keywords:** hydrothermal treatment, rigid PVC plastics (R-PVC), dechlorination, CaCO_3_ migration mechanism

## Abstract

Rigid PVC plastics (R-PVC) contain large amounts of chlorine, and improper disposal can adversely affect the environment. Nevertheless, there is still a lack of sufficient studies on hydrothermal treatment (HTT) for the efficient dechlorination of R-PVC. To investigate the migration mechanism of chlorine during the HTT of R-PVC, R-PVC is treated with HTT at temperatures ranging from 220 °C to 300 °C for 30 min to 90 min. Hydrochar is characterized via Fourier transform infrared spectrometry and X-ray photoelectron spectroscopy. The results revealed that the hydrothermal temperature is the key factor that affects the dechlorination of R-PVC. Dramatic dechlorination occurs at temperatures ranging from 240 °C to 260 °C, and the dechlorination efficiency increases with the increase in the hydrothermal temperature. The main mechanism for the dechlorination of R-PVC involves the nucleophilic substitution of chlorine by -OH. CaCO_3_ can absorb HCl released by R-PVC and hinder the autocatalytic degradation of R-PVC; hence, the dechlorination behavior of R-PVC is different from that of pure PVC resins. Based on these results, a possible degradation process for R-PVC is proposed. This study suggests that HTT technology can be utilized to convert organochlorines in R-PVC to calcium chloride, achieving the simultaneous dechlorination of R-PVC and utilization of products.

## 1. Introduction

Owing to the energy crisis and environmental degradation, plastic waste as the major component of solid waste has attracted increasing attention [1,2,3]. Among all plastics, polyvinyl chloride (PVC) has been widely used in construction (24.6%), packaging (19.5%), agriculture (15.5%), daily consumption (30.4%), and other fields (10%) due to its excellent performance and cost-effectiveness [4]. PVC has become one of the five general-purpose plastics after polyethylene and polypropylene [5]. PVC products are mainly divided into soft and rigid products, where rigid PVC accounts for ~78% of all PVC products [6]. In 2016, the global production of PVC had reached 58.5 million tons, with an expected annual growth rate of 3.2% [7]. Even though R-PVC products are more durable than other plastics, they eventually become plastic waste.

PVC comprises vinyl chloride (VC) via free-radical addition polymerization, with a theoretical chlorine content of ~57% [8]. Owing to the limitation of separation technology, it is difficult to effectively separate PVC from a mixture of waste plastics, thereby leading to the increase in the chlorine content of plastic waste [7,9]. During waste incineration, HCl released by PVC is close to half of the total amount, which is the main source of organic chlorine in plastic waste, adversely limiting the safety and resource utilization of plastic waste, and its disposal poses the main environmental risk [10]. Although pyrolysis may be appropriate for converting PVC waste into energy, a typical problem with the pyrolysis of PVC is that the resulting product usually contains large amounts of chlorinated compounds that are difficult to use directly [11]. Therefore, it is imperative to find an effective method to remove chlorine from PVC before combustion or pyrolysis.

In recent years, hydrothermal treatment technology (HTT) has been considered as an effective and environmentally friendly method for removing chlorine from PVC. HTT is a simple and rapid reaction, with no heat or mass transfer limitations and excellent process efficiency [12,13]. Studies have reported that the HTT of pure PVC resin can effectively break the C–Cl bond in PVC, and through elimination and nucleophilic substitution of Cl by -OH, the organochlorine in the PVC resin can be converted into HCl or chloride ions that can be removed [14,15]. Nevertheless, a previous study mainly focused on the dechlorination of a pure PVC resin. Owing to the defects inherent to the structure of PVC resin, it is often difficult to use alone. During production, lead salt, metal soap, organic tin, and other stabilizers, as well as a large number of CaCO_3_ fillers, are typically added to meet the requirements for applications and reduce production costs [16]. Results revealed that CaCO_3_ can absorb the HCl released during the pyrolysis of PVC and prevent the autocatalytic degradation of PVC [16,17]. However, there is a lack of pertinent research investigating the potential impact of CaCO_3_ on PVC dechlorination under subcritical conditions. Alkali, metal oxides, and inorganic salts can significantly improve the dechlorination efficiency of a PVC resin and affect the migration behavior of chlorine under subcritical conditions [14,18,19]. Qi [6] investigated the dechlorination behavior of an infusion tube (i.e., flexible PVC) and a urine sample collector (i.e., rigid PVC) in methanol, and the results revealed that the plasticizer in the infusion tube promotes the dechlorination of PVC, while the heat stabilizer in the urine sample collector limits dechlorination. Yoshioka [20,21] focused on the dechlorination behavior of a pure PVC resin and flexible PVC plastic in subcritical water and found that HCl is removed by the pure PVC resin and flexible PVC plastic via elimination and -OH substitution, respectively. Thus, pure PVC resin and PVC plastic exhibit different dechlorination behaviors. Therefore, the reaction mechanism of a pure PVC resin in subcritical water cannot be used to characterize the dechlorination and degradation behaviors of PVC plastics. Nevertheless, the dechlorination behavior of rigid PVC, which often contains a substantial amount of filler, has not been addressed. This aspect holds particular significance for deciphering the dechlorination mechanism of actual PVC solid waste, given that rigid PVC constitutes a predominant proportion of such waste.

Currently, studies on the Cl migration behavior of rigid polyvinyl chloride (R-PVC) under HTT conditions are still lacking. In this study, the dechlorination of R-PVC in subcritical water is investigated, as well as the effect of the hydrothermal temperature and residence time on the dechlorination efficiency of R-PVC, the migration behavior of Cl in HTT, and the dechlorination mechanism of R-PVC, which are key to promoting the application of HTT for the disposal of plastics and realizing high-value utilization of the products.

## 2. Materials and Methods

### 2.1. Experiment Materials

A PVC electrical wire groove (Ji Cheng Plastic Co., Ltd., Hangzhou, China) was the R-PVC used in this experiment. After grinding the groove with a crusher, it was passed through an 80-mesh sieve to obtain PVC particles with a size of less than 2 mm to obtain a uniform-sized material. Next, the material was dried in an oven at 105 °C for 24 h and stored in a sealed bag.

### 2.2. Experimental Method

#### 2.2.1. Hydrothermal Treatment Process

HTT experiments were conducted in a 500 mL autoclave. The HT-500FJ autoclave is a flanged mechanical reaction kettle produced by Shanghai Huotong Experimental Instrument Co., Ltd., Shanghai, China. The body of the reaction kettle is composed of 316 stainless steel, which was equipped with a magnetic coupling seal and mechanical stirring to ensure sufficient stirring during the experiment, and the speed can be controlled from 0 to 1500 rpm. The maximum operating temperature of the reactor is 300 °C, and the maximum operating pressure can reach 22.5 MPa. A K-type thermocouple was used to measure temperature, which was equipped with a temperature control system, and the temperature was accurately controlled at ±1 °C.

**Figure 1:**
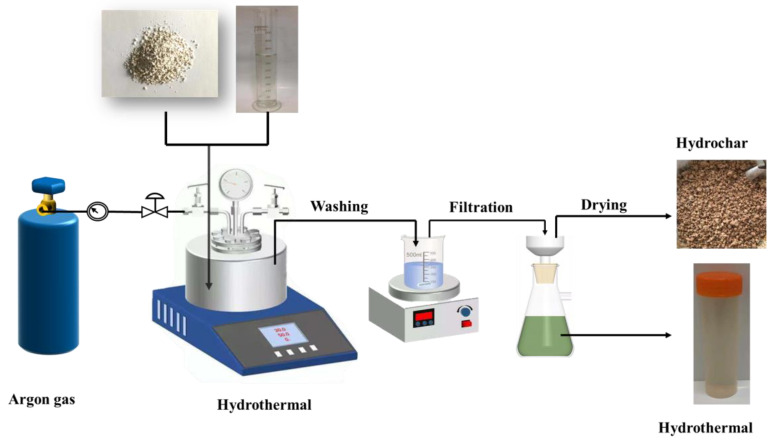
The schematic diagram of hydrothermal treatment.

Figure 1 shows the schematic diagram of hydrothermal treatment. First, 35 g of an R-PVC sample and 350 mL of deionized water were added into the reactor, and the reactor was sealed. Dechlorination with a solid–liquid ratio of 1:10 can make the volume of water not a restricting factor for dichlorination [22,23]. Before each experiment, the argon cylinder valve was opened, as well as the argon purge inlet and outlet valves of the reactor. Argon is 99.99% pure. The reactor and pipeline were purged with argon flowing at ~300 mL/min for 10 min; then, the reactor inlet valve, outlet valve, pipe valve, and argon cylinder valve were closed in the order mentioned to ensure that the reactor was in the closed state. Next, the power supply was switched off, the hydrothermal reaction temperature and residence time were set, and the stirring speed was set to 200 rpm/min. The retention time is the time when the reactor is kept at a predetermined temperature except for preheating and cooling times. Table 1 summarizes the reaction conditions of the hydrothermal experiment. After the reaction was completed, the reactor was cooled to room temperature by cooling water outside the reactor. The pressure-reducing valve was opened, and the gas product rapidly escaped. The cover of the reactor was opened, the solid–liquid mixture in the reactor was poured into the beaker, the reactor was washed thrice with deionized water, and the washing liquid was poured into the beaker. The mixture was filtered under vacuum using a Brønsted funnel, and the solid products were washed thrice using a magnetic agitator to remove the soluble matter attached to the solid. The separated solid product was dried at 105 °C for 24 h in a blast oven, which was hydrochar, ground to a particle size of less than 74 µm (200 mesh) using a sample mill, and stored in a sealed bag for testing and analysis. The filtrate and washing liquid were collected and poured into a volumetric flask with a constant volume of 1000 mL, which was hydrothermal. For the convenience of analysis and discussion, the product is abbreviated as “XX-XX-XX.” For example, S-240-60 and L-240-60 represent the hydrochar and hydrothermal products obtained at a hydrothermal temperature of 240 °C and a residence time of 60 min. All experiments were conducted in triplicate to ensure reproducibility and consistency.

#### 2.2.2. Characterization of Products

**Figure 2:**
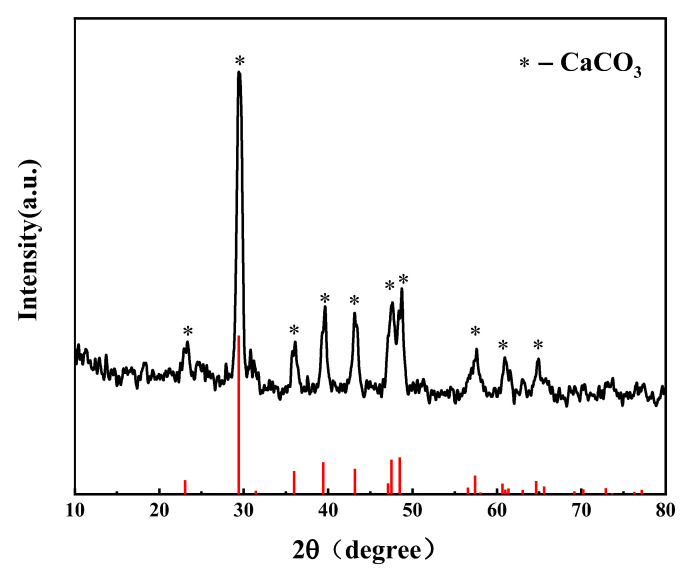
X-ray diffraction pattern of PVC.

Elements C, H, N, and S in R-PVC were determined using an elemental analyzer (EA300, Euro Vector Company, Foggia, Italy). The mass percentage of O was obtained via subtraction. The ash content was determined using an industrial analyzer (SDLA718, Hunan Sunde Company, Xingsha, China), and chlorine and other elements were determined via X-ray fluorescence spectrometry (XRF). Table 2 summarizes the elemental and ash analyses of R-PVC. The X-ray fluorescence spectrometer can be used for the qualitative and quantitative analysis of the elements with an atomic number greater than 9 in solid materials, and the analysis results are related to oxygen and ash [15,18]. Fourier-transform infrared spectroscopy (Nicolet 6700, Thermo Nicolet Co., Waltham, MA, USA) was employed to characterize the surface functional groups of the hydrochar. The chemical forms of C and Cl in the R-PVC and hydrochar were analyzed via X-ray photoelectron spectroscopy (XPS). XPS peaks were divided using Thermo Vantage software v5.9931. The C1s electron binding energy (284.8 eV) was used as the internal standard to correct the charge potential shift, and the Gauss–Lorentz formula was used to fit the XPS peaks of C and Cl. X-ray diffractometry (XRD) was employed to analyze the major metallic compounds contained in R-PVC. Figure 2 shows the analysis results. CaCO_3_ is the main compound in R-PVC used in the experiment. The chloride ion concentration in the hydrothermal reaction was determined via ion chromatography; hence, the chloride in the hydrothermal reaction can be defined as inorganic chlorine. As chlorine in R-PVC mainly exists as C–Cl bonds, chlorine in R-PVC is defined as organochlorine. The calcium ion concentration in the hydrothermal reaction was determined via inductively coupled plasma mass spectrometry (ICP-MS, PE NexION series).

#### 2.2.3. Data Analysis Method

After HTT, the hydrochar yield (X_0_), dechlorination efficiency (DE) of R-PVC, and the conversion rate of organochlorine (C) were calculated according to Formulae (1), (2), and (3), respectively. M_i_ represents the mass of residual solid (hydrochar) after HTT, g; M_0_ represents the mass of the R-PVC sample added to the reaction vessel before the HTT experiment, g; m_Cl-0_ represents the concentration of Cl in R-PVC, wt%; m_Cl-i_ denotes the Cl concentration of hydrochar after HTT, wt%; and I_Cl-i_ is the Cl mass of hydrothermal, g. By using the chlorine content of hydrochar to calculate the dechlorination efficiency of R-PVC, the effect of the loss through hydrothermal collection on the calculation of dechlorination efficiency can be reduced.

The total mass loss rate of the solid in R-PVC hydrothermal treatment is W, and the mass loss rates of Cl and Ca in R-PVC are W_Cl_ and W_Ca_, respectively, calculated according to Formulae (4), (5), and (6), respectively. m_Ca-0_ denotes the Ca concentration of R-PVC, wt%, and m_Ca-i_ represents the Ca concentration of hydrochar after the hydrothermal reaction, wt%.

(1) $$\mathrm{Hydrothermal}\;\mathrm{carbon}\;\mathrm{yield}\left(X_{0}\right)=\left(M_{i}/M_{0}\right)\times 100\%$$

(2) $$\mathrm{Dechlorination}\;\mathrm{efficiency}\left(\mathrm{DE}\right)=\left(M_{0}\times m_{cl-0}-M_{i}\times m_{cl-i}\right)/(M_{0}\times m_{\mathrm{cl}-0})\times 100\%\;$$

(3) $$\mathrm{Organochlorine}\;\mathrm{conversion}\;\mathrm{rate}\left(C\right)=I_{\mathrm{Cl}-i}/(M_{0}\times m_{cl-0})\times 100\%$$

(4) $$\mathrm{Total}\;\mathrm{mass}\;\mathrm{loss}\;\mathrm{rate}\left(W\right)=M_{0}-M_{i}/M_{0}\times 100\%$$

(5) $$\mathrm{The}\;\mathrm{mass}\;\mathrm{loss}\;\mathrm{rate}\;\mathrm{of}\;\mathrm{Cl}\left(W_{\mathrm{Cl}}\right)=(M_{0}\times m_{\mathrm{cl}-0}-M_{i}\times m_{\mathrm{cl}-i})/M_{0}\times 100\%$$

(6) $$\mathrm{The}\;\mathrm{mass}\;\mathrm{loss}\;\mathrm{rate}\;\mathrm{of}\;\mathrm{Ca}\left(W_{\mathrm{Ca}}\right)=(M_{0}\times m_{\mathrm{Ca}-0}-M_{i}\times m_{\mathrm{Ca}-i})/M_{0}\times 100\%$$

## 3. Results and Discussion

### 3.1. Effect of Reaction Parameters on the Dechlorination Efficiency of R-PVC

**Figure 3:**
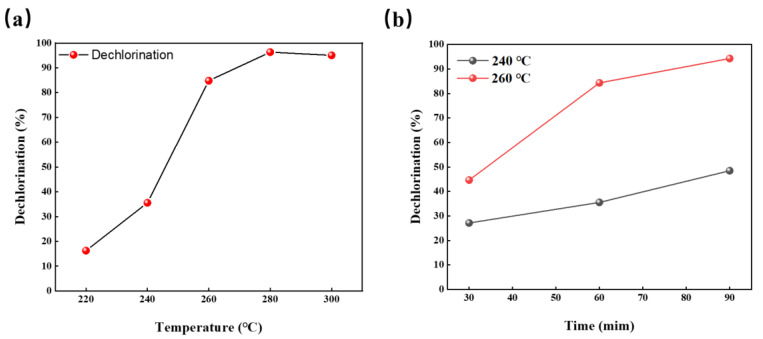
(**a**) Change in the dechlorination efficiency of R-PVC as a function of the hydrothermal temperature at a residence time of 60 min. (**b**) Change in the dechlorination efficiency of R-PVC as a function of the residence time at hydrothermal temperatures of 240 °C and 260 °C.

The hydrothermal temperature and residence time are key external factors that affect the dechlorination of a PVC resin. Several research results revealed that the reaction temperature is the key factor that affects the dechlorination of the PVC resin [14,22,24]. To examine the dechlorination behavior of R-PVC under different reaction conditions, the dechlorination efficiency of R-PVC at different hydrothermal temperatures and residence times was investigated. Figure 3a shows the trend of the dechlorination efficiency of R-PVC with the change in the hydrothermal temperature at a residence time of 60 min. As can be observed from Figure 3a, in the range of hydrothermal temperatures between 220 °C and 280 °C, the dechlorination efficiency of R-PVC increased with the increase in the hydrothermal temperature, rapidly increasing from 16.23% to 96.32% and reaching the maximum. The increase in the dechlorination efficiency revealed an increasing trend first and then a decreasing trend. With the increase in the hydrothermal temperature from 220 °C to 240 °C, the dechlorination efficiency of R-PVC increased by 19.3%. With the increase in the hydrothermal temperature to 260 °C, the increase in the dechlorination efficiency reached the maximum. With the continuous increase in the hydrothermal temperature to 280 °C, the increase in the R-PVC dechlorination efficiency was significantly reduced, with an increase of only 11.51%, indicating that at a hydrothermal temperature of 240 °C, R-PVC starts to undergo drastic dechlorination and that the main dechlorination process occurs in the range of 240–260 °C. Therefore, the hydrothermal temperature is a key factor that affects the dechlorination efficiency and reaction rate of R-PVC. With the increase in the hydrothermal temperature, the saturated vapor pressure in the closed reactor increased, and under hydrothermal conditions of high temperature and high pressure, the C–Cl bond in R-PVC molecules was broken, and hydrolysis was promoted. In addition, the physical properties of water under high temperature and pressure conditions also changed significantly, the ionization constant decreased, the ion product became larger, and the polarity of water molecules became weaker, making the properties of water similar to organic solvents and promoting the mutual solubility of water molecules and R-PVC. Notably, when the hydrothermal temperature reached 300 °C, the dechlorination efficiency of R-PVC exhibited an extremely small decrease from 96.32% to 96.05% because the hydrochar formed when R-PVC reaches a high dechlorination efficiency can re-absorb Cl^−^ in the hydrothermal treatment, thus reducing the dechlorination efficiency.

Prolonging the residence time at a certain hydrothermal temperature is another key factor that promotes PVC dichlorination. Figure 3b shows the change in the dechlorination efficiency of R-PVC with the residence time at different hydrothermal temperatures. At hydrothermal temperatures of 240 °C and 260 °C, with the increase in the retention time from 30 min to 90 min, the dechlorination efficiency of R-PVC increased from 27.17% and 44.67% to 44.64% and 94.27%, respectively, indicating that although the dechlorination temperature of R-PVC is reached, a certain reaction time is still needed to achieve a high dechlorination efficiency. Therefore, a prolonged residence time can promote the dechlorination of R-PVC and improve the dechlorination efficiency. Poerschmann et al. [25] have investigated the dechlorination behavior of a pure PVC resin in subcritical water at 180–260 °C. The results revealed that at a hydrothermal temperature of 235 °C and a residence time of 15 h, the dechlorination efficiency of a PVC resin could reach 99%, indicating that the extension of the residence time can promote the dechlorination of the PVC resin. However, with the increase in the residence time, the increase rate of the R-PVC dechlorination efficiency at 260 °C was 49.63%, which was significantly greater than that (21.33%) at 240 °C. Therefore, the effect of residence time on dechlorination efficiency varies with the hydrothermal temperature. The dechlorination efficiency of R-PVC at the same residence time at 260 °C was greater than that at 240 °C, indicating that the effect of hydrothermal temperature on the dechlorination efficiency of R-PVC is greater than that of the residence time. The above results revealed that, although the extended residence time can compensate for the lack of temperature to some extent, the hydrothermal temperature is still a key factor that affects the dechlorination of R-PVC.

### 3.2. Chlorine Balance

**Figure 4:**
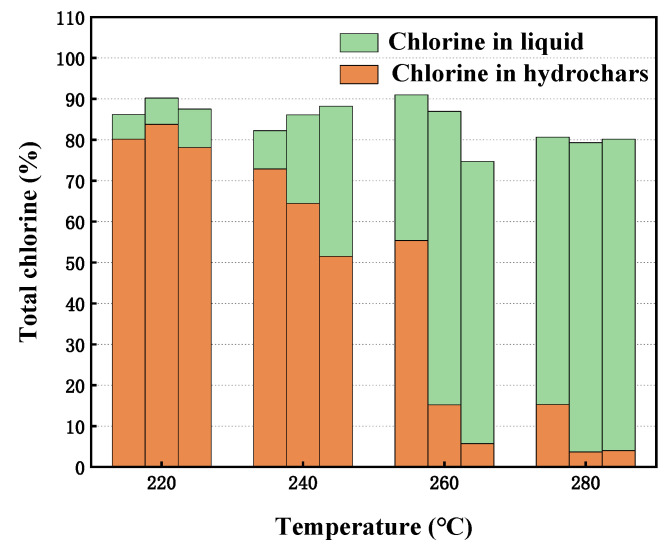
Distribution of Cl in HTT at different hydrothermal temperatures for 30 min, 60 min, and 90 min.

To verify whether the organochlorine in R-PVC is completely converted into inorganic chlorine during hydrothermal treatment, the Cl content in hydrochar and hydrothermal treatment was measured. Figure 4 shows the chlorine balance of R-PVC dechlorination under different hydrothermal conditions. Studies have reported that under subcritical hydrothermal conditions, HCl in the pure PVC resin can be removed via elimination or nucleophilic substitution of Cl with H_2_O as the nucleophile [19,24,26,27]. With the increase in the hydrothermal temperature and residence time, the content of organochlorine in hydrothermal carbon decreased, while the content of inorganic chlorine (Cl^−^) in hydrothermal treatment increased, indicating that HTT can effectively remove Cl in R-PVC and convert organochlorine into inorganic chlorine that is soluble in water. The sum of the chlorine content in the hydrochar and hydrothermal treatment was not 100%, and the total chlorine loss increased with the increase in the hydrothermal temperature. Takeshita [28] and Zhao [27] have reported that with the increase in the hydrothermal temperature, the mass of hydrochar decreases and the mass of the produced gas increases, but chlorine is not detected in the gas. Therefore, with the increase in the temperature, the total chlorine loss increases due to the detection error of different instruments and the loss during sample collection.

### 3.3. Chlorine Migration Behavior

#### 3.3.1. Fourier-Transform Infrared Spectroscopy

**Figure 5:**
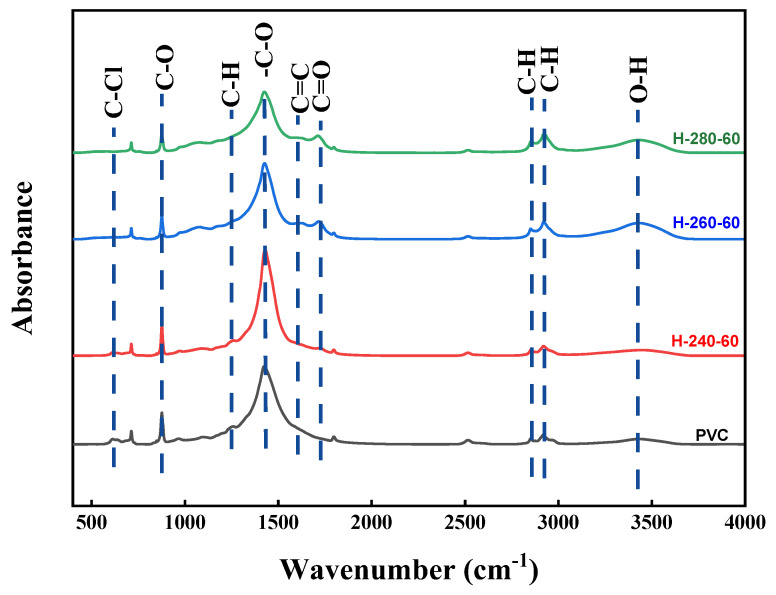
FTIR spectra of R-PVC and hydrochar obtained via HTT at 240 °C, 260 °C, and 280 °C for 60 min.

Through the infrared spectral analysis of R-PVC and hydrochar, the functional group information of R-PVC and hydrochar samples can be obtained, which is beneficial for understanding the dechlorination mechanism. Figure 5 shows the infrared spectra of R-PVC and hydrochar obtained at different hydrothermal temperatures at a residence time of 60 min. Different hydrothermal temperatures led to significant changes in the surface functional groups of hydrochar. The absorption peak at 614 cm^−1^ corresponded to the stretching vibration of C–Cl bonds, and the absorption peak at 1257 cm^−1^ corresponded to the vibration of C–H bonds in CH-Cl bonds. Absorption peaks at 2850 cm^−1^ and 2918 cm^−1^ were assigned to C-H asymmetric and symmetric stretching from -CH_2_- in -CH_2_-CHCl-. The absorption peaks at 1427 cm^−1^, 876 cm^−1^, and 712 cm^−1^ corresponded to the tensile vibration, out-of-plane, and in-plane bending vibrations of the C–O bond in CaCO_3_, respectively. The absorption peaks at 3429 cm^−1^ corresponded to the O–H stretching vibrations. The absorption peaks at 1624 cm^−1^ and 1712 cm^−1^ corresponded to the C=C skeleton vibrations of the benzene ring plane and stretching vibrations of the C=O bond in adipose ketones, respectively [29,30]. With the increase in the hydrothermal temperature, the intensities of the two absorption peaks at 614 cm^−1^ and 1257 cm^−1^ weakened and almost disappeared at a hydrothermal temperature of 260 °C, indicating that the C–Cl bond breaks with the increase in the hydrothermal temperature. At the same time, the intensity of the absorption peak at 3429 cm^−1^ increased significantly at a hydrothermal temperature of 260 °C, but decreased significantly with the increase in the hydrothermal temperature to 280 °C, indicating that the main reaction is the substitution of -Cl by -OH at 260 °C, while dehydration is the main reaction mechanism at a hydrothermal temperature of 280 °C. At a hydrothermal temperature of 280 °C, the absorption peak intensity at 1712 cm^−1^ reached the maximum, also verifying that -OH is removed through dehydration. At a hydrothermal temperature of 260 °C, a new absorption peak was observed at 1624 cm^−1^, which increased with the increase in the hydrothermal temperature, indicating that hydrothermal carbon undergoes aromatization and that the aromatization degree increases with the increase in the hydrothermal temperature. The absorption peaks at 2918 cm^−1^ and 2850 cm^−1^ shifted to the right to 2923 cm^−1^ and 2853 cm^−1^, indicating that the absorption peaks at 2923 cm^−1^ and 2853 cm^−1^ do not correspond to the methylene group in original R-PVC, but are generated during aromatization after HCl removal. The significantly weakened absorption peaks at 1427 cm^−1^, 876 cm^−1^, and 712 cm^−1^ indicated that the CO_3_^2−^ content decreases and the degradation of CaCO_3_ slows down. According to the above analysis, R-PVC is speculated to mainly remove HCl via OH substitution, the released HCl is captured by CaCO_3_, and CaCl_2_ is formed for attachment on the hydrochar surface or dissolved in the hydrothermal reaction. Tongamp et al. [15] have employed XRD to analyze the co-grinding products of oyster shells and a PVC resin, and the results revealed that CaCO_3_ exhibits a strong ability to capture HCl released by the PVC resin. The specific reaction mechanism is expressed in Formula (7).

(7) $$\left[-{\mathrm{CH}}_{2}-\mathrm{CHCl}-\right]+{\mathrm{CaCO}}_{3}\to \left[-{\mathrm{CH}}_{2}-\mathrm{CHOH}-\right]+{\mathrm{CO}}_{2}+{\mathrm{CaCl}}_{2}\;$$

#### 3.3.2. X-ray Photoelectron Spectroscopy

**Figure 6:**
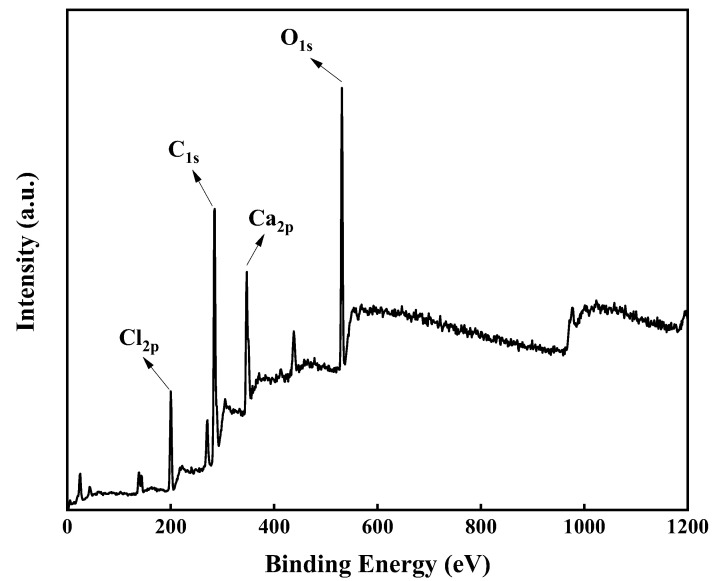
XPS wide-scan profile of R-PVC.

XPS was mainly utilized to analyze the surface composition of R-PVC and hydrochar, which is a powerful tool to provide information on the relative number of different functional groups on solid surfaces. To further reveal the dechlorination behavior of R-PVC under HTT, XPS profiles of R-PVC and hydrochar were recorded. Figure 6 shows the high-resolution XPS spectra of C1s, O1s, Cl2p, and Ca2p in R-PVC. The peaks of C1s, O1s, Cl2p, and Ca2p in R-PVC were clearly observed, indicating that the R-PVC surface is mainly composed of carbon, oxygen, chlorine, and calcium. The sum of the peak areas of the four elements in R-PVC and hydrochar was recorded as 100%, and the change in the relative ratio of the peak areas of each element was compared to examine the change trend of the surface elements during the HTT of R-PVC.

Table 3 summarizes the data obtained from the surface element analysis of the R-PVC sample at a residence time of 60 min and at hydrothermal temperatures of 240 °C and 280 °C. By comparing the results shown in Table 3, although the total elemental composition of the sample did not change significantly, the chemical composition of the surface changed significantly. With the increase in the hydrothermal temperature, the relative proportion of C in the R-PVC surface elements increased from 63.14% to 75.06%, while the content of Ca decreased from 6.72% to 2.45%, and the contents of O and Cl increased first and then decreased. With the increase in the hydrothermal temperature to 240 °C, the contents of O and Cl increased from 22.33% and 7.81% to 22.39% and 9.22%, respectively. With the continuous increase in the hydrothermal temperature to 280 °C, the contents of O and Cl decreased to 18.87% and 2.45%, respectively. Clearly, according to the changes in the composition of the above elements, the functional groups on the R-PVC surface were confirmed to change with the increase in the hydrothermal temperature. The increase in the C content indicated that the carbonization of R-PVC occurs during HTT, which is a process of carbon accumulation. Combined with the above infrared spectral analysis results, the decrease in the Ca content corresponded to the degradation of CaCO_3_ in R-PVC during HTT. At a hydrothermal temperature of 240 °C, the Cl content increased significantly. The HCl released by R-PVC is speculated to be captured by CaCO_3_ and adhered to the solid surface. In the initial stage of pyrolysis, molecular isomerization also possibly occurred inside R-PVC, affording isomeric monomers with the Cl atom outside the molecule. Gui [31,32] investigated the pyrolysis mechanism of PVC using a metal mesh reactor and revealed that molecular isomerization occurs in PVC in the initial stage of pyrolysis and that the Cl atom in the formed isomerized monomer is outside the molecule. The increase in the O content corresponded to the strengthening of -OH substitution. With the increase in the hydrothermal temperature, the contents of Cl and Ca decreased simultaneously. Combined with the decrease in the CO_3_^2−^ content in the previous analysis, this result indicated that the CaCO_3_ filler participates in the dechlorination of R-PVC and generates water-soluble metal chloride CaCl_2_, which is transferred from hydrochar to a hydrothermal product.

**Figure 7:**
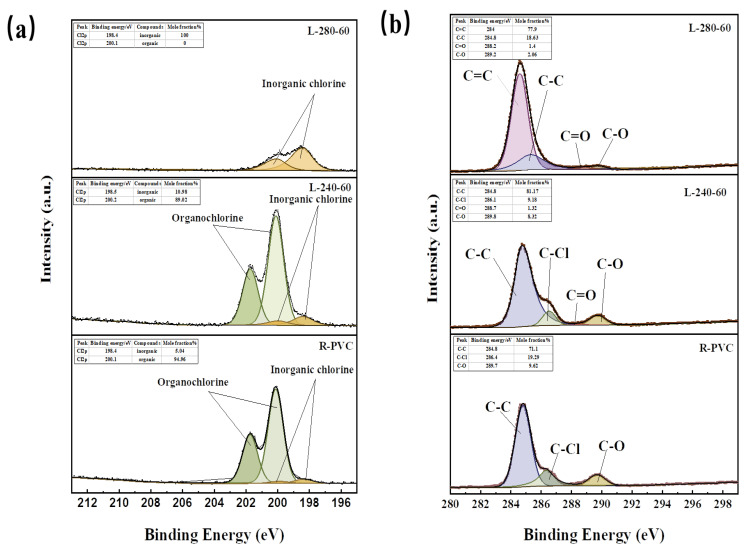
(**a**) Cl 2p XPS profiles of R-PVC and hydrochar from HTT at 240 °C and 280 °C for 60 min. (**b**) C1s XPS profile with curve fitting of R-PVC and hydrochar from HTT at 240 °C and 280 °C for 60 min.

To understand the migration behavior of chlorine in R-PVC during HTT, it is crucial to analyze the forms in which Cl occurs during migration. Based on this result, the Cl2p energy spectrum peaks of R-PVC and the hydrochar obtained at 240 °C and 280 °C at a residence time of 60 min were further analyzed. According to the method described in Section 2.2.2, the peaks observed in the Cl 2p energy spectrum were divided into peaks. Figure 7a shows the fitting results. The occurrence ratio of corresponding substances according to peak area was calculated. Almost all of the Cl in R-PVC was organochlorine, accounting for ~94.96%. With the increase in the hydrothermal temperature to 240 °C and 280 °C, the organic chlorine content of the hydrothermal carbon decreased to 89.02% and 0%, respectively, while the inorganic chlorine content increased to 10.98% and 100%, respectively, indicating that organic chlorine in R-PVC can be converted into inorganic chlorine via HTT. However, after washing, some inorganic chlorine was still present in the hydrochar. At a hydrothermal temperature of 280 °C, all of the chlorine remaining on the hydrothermal carbon surface as shown in Figure 7a was inorganic chlorine, indicating that it is possible to further remove the Cl from hydrothermal carbon by specific extraction or leaching techniques.

To better examine the migration behavior of Cl in R-PVC during HTT, the C1s energy spectrum peaks of R-PVC and the hydrochar obtained at 240 °C and 280 °C at a residence time of 60 min were further analyzed. Figure 7b shows the results of C1s peak separation fitting. R-PVC mainly consisted of C–C, C–Cl, and C–O bonds, with bond energies of 284.8 eV, 286.4 eV, and 289.7 eV, respectively. Based on the infrared spectral analysis of the hydrochar, it consisted of C=O and C=C bonds. At a hydrothermal temperature of 240 °C, the relative peak areas of the C–Cl and C–O bonds decreased from 19.36% and 9.58% to 9.19% and 8.32%, respectively, replaced by the newly generated C=O bond. Thus, R-PVC mainly removes Cl via -OH substitution, and dehydration subsequently occurs to generate C=O bonds to form a fatty ketone structure. With the increase in the hydrothermal temperature to 280 °C, C–Cl bonds completely disappeared, the relative peak area of the C–C bond significantly decreased, and the relative peak volume of the newly generated C=C bond was 77.90%, indicating that the carbon framework of R-PVC is broken and that aromatization occurs within the molecule to form an aromatic hydrocarbon structure.

### 3.4. Mass Balance of R-PVC

**Figure 8:**
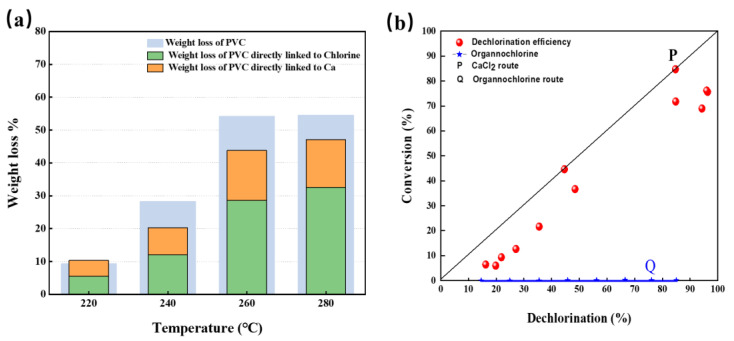
(**a**) Variation in the mass loss rate (W) of R-PVC, mass loss rate (W_Cl_) of Cl, and mass loss rate (W_Ca_) of Ca in R-PVC as a function of temperature. (**b**) Relationship between the dechlorination efficiency of R-PVC and organochlorine conversion rate.

The mass loss rate (W) of R-PVC, mass loss rate of Cl (W_Cl_), and mass loss rate of Ca (W_Ca_) in R-PVC varied with temperature at hydrothermal temperatures ranging from 220 °C to 280 °C and at a reaction time of 60 min, and are shown in Figure 8a. With the increase in temperature, W, W_Cl_, and W_Ca_ increased. With the increase in the hydrothermal temperature, W_Cl_ increased from 5.48% to 32.56% and W_Ca_ increased from 4.85% to 15.11%, indicating that the removal of Cl and Ca from R-PVC is the main cause of R-PVC’s weightlessness. Combined with the above analysis, the reduction in Cl content in R-PVC was the main cause of CaCO_3_ degradation. CaCO_3_ captured the Cl released by R-PVC and generated CaCl_2_ and CO_2_, leading to the quality loss of R-PVC. Except W_Cl_ and W_Ca_, ~10% of the weight loss in R-PVC corresponded to the release of CO_2_.

Figure 8b shows the relationship between the dechlorination efficiency of R-PVC and the conversion efficiency of organochlorine. Migration of Cl in R-PVC occurred via two routes: (1) Dll of the removed HCl was captured by CaCO_3_ to form metal chloride CaCl_2_, which can be represented by line P. (2) The removed chlorine was released as HCl or volatile organochlorine compounds, which can form a line equal to the X-axis (denoted by line Q). The dechlorination efficiency (DE) and organochlorine conversion rate (C) of R-PVC were close to line P, indicating that most of the removed chlorine exists as metal chloride CaCl_2_. Combined with the reduction in CO_3_^2−^ and Ca content in the infrared spectral analysis and XPS analysis mentioned above, CaCO_3_ can combine with HCl released by R-PVC to form metal chloride CaCl_2_.

### 3.5. Dechlorination Mechanism of R-PVC

**Figure 9:**
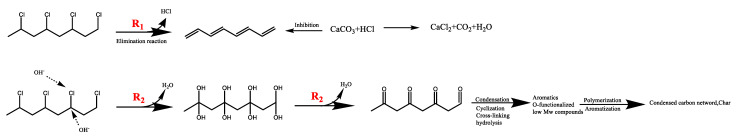
Chlorine migration and transformation during the hydrothermal treatment of R-PVC.

According to the above experimental results and analysis, the migration and transformation path of chlorine in the hydrothermal treatment of R-PVC is shown in Figure 9. On the one hand, with the increase in the hydrothermal temperature, the temperature of R-PVC increased, and the initial chain dehydrogenation caused by the structural defects of R-PVC started, as indicated by the R_1_ route. However, the autocatalysis of R-PVC was inhibited by the high content of CaCO_3_ in R-PVC, which can combine with the released HCl, thereby preventing the formation of polyene structures; hence, the presence of conjugated double bonds was not detected during the hydrothermal process. On the other hand, with the increase in the hydrothermal temperature, the concentration of -OH radicals in water increased considerably, thereby promoting the substitution of -OH to -Cl. The presence of CaCO_3_ promoted the substitution of -H on the C atom attached to -OH for the continuous replacement by -OH to form a double OH structure, which was extremely unstable and underwent rapid dehydration to form more stable aliphatic ketones, as indicated by the R_2_ route. Subsequent aromatization and polymerization afforded dense, cross-linked hydrothermal carbon. Therefore, a large number of C=C bond functional groups were detected at a hydrothermal temperature of 280 °C. In summary, in the hydrothermal process, R-PVC mainly removes HCl via -OH substitution.

## 4. Conclusions

Hydrothermal treatment can effectively remove Cl from R-PVC. With the increase in the hydrothermal temperature and residence time, the dechlorination efficiency increases. The hydrothermal temperature is the most critical factor that affects the dechlorination efficiency of R-PVC. CaCO_3_ can absorb the HCl released during the pyrolysis of R-PVC, thus inhibiting the autocatalytic degradation of R-PVC. R-PVC mainly removes HCl via the nucleophilic substitution of Cl by -OH, and the removed HCl is captured by CaCO_3_ to generate CaCl_2_ and transferred to a hydrothermal product. The residual chlorine in hydrochar mainly exists as inorganic chlorine, which can be removed through further washing or leaching. Therefore, the presence of a CaCO_3_ filler affects the migration behavior of chlorine in R-PVC and changes the degradation products. The conversion of Cl from R-PVC to metal chlorides in the hydrothermal reactor is more advantageous than the recovery of HCl. In particular, in commercial factories, the corrosion of pipes and equipment by HCl as well as atmospheric pollution can be considerably reduced. The obtained metal salt can provide the basis for the subsequent catalytic conversion to chlorine gas, thus improving the utilization value of the product and realizing the high-value utilization of chlorine. Combined with the dichlorination conversion path of rigid PVC presented herein, further research can focus on the selection of appropriate catalysts to improve the dechlorination efficiency of R-PVC in solid waste at lower hydrothermal temperatures. In conclusion, this paper provides a theoretical basis for the process design and parameter optimization of MSW dechlorination.

## Figures and Tables

**Table 1:** Experimental parameters.

Parameters	Values
Temperature (°C)	220, 240, 260, 280,3 00
Residence time (min)	30, 60, 90

**Table 2:** Elemental analysis and ash analysis of R-PVC plastics.

PVC	A_d_	C	H	N	S	O ^a^	Cl	Ca	Pb	Mg	Ti	Si	Fe	Sr	P
wt%	37.27	26.60	2.50	nd	nd	33.63	33.80	28.14	0.50	0.50	0.40	0.07	0.07	0.02	0.01

^a^ difference: O% = 100%-C%-H%-N%-A_d_%. nd: not detect. d: dry basis.

**Table 3:** Surface energy spectrum analysis of R-PVC and hydrochar at different hydrothermal treatment temperatures (wt%, db).

Samples	C	O	Cl	Ca
R-PVC	63.14	22.33	7.81	6.72
240 °C	63.19	22.39	9.22	5.21
280 °C	75.06	18.87	3.63	2.45

## Data Availability

Not applicable.
